# HPV-Related Promoter Methylation-Based Gene Signature Predicts Clinical Prognosis of Patients With Cervical Cancer

**DOI:** 10.3389/fonc.2021.753102

**Published:** 2021-10-21

**Authors:** Ran Zhou, Zhuo Chen, Zuo-Run Xiao, Shou-Li Wang, Chao Rong

**Affiliations:** ^1^Department of Pharmacy, The First Affiliated Hospital of USTC, Division of Life Sciences and Medicine, University of Science and Technology of China, Hefei, China; ^2^Department of Pathology, The First Affiliated Hospital of USTC, Division of Life Sciences and Medicine, University of Science and Technology of China, Hefei, China; ^3^Department of Pathology, School of Biology & Basic Medical Sciences, Soochow University, Suzhou, China

**Keywords:** cervical cancer, human papillomavirus (HPV), DNA methylation, prognostic biomarkers, gene signature

## Abstract

Persistent high-risk HPV infection drives tumorigenesis in various human malignancies, including cervical, oropharyngeal, anal, and vulvar carcinomas. Although HPV-related tumors arise in several different sites, they share many common genetic and epigenetic events. Complex and heterogeneous genomic aberrations and mutations induced by high-risk HPV contribute to the initiation and progression of cervical cancer (CC). However, the associations between high-risk HPV infection and DNA methylation have not been clearly investigated. In the present study, HPV-related gene promoter methylation signature was comprehensively analyzed using multiple interactive platforms. CC patients were successfully classified into high-risk and low-risk groups with significant differences in clinical outcomes based on the HPV-related gene promoter methylation signature. Moreover, the protein levels of ALDH1A2 and clinical prognostic value were confirmed in the CC patients cohort. In summary, our study provides compelling evidence that HPV-related gene promoter methylation signature serves as a strong prognostic signature for CC patients. Clinical investigations in large CC patient cohorts are greatly needed to pave the way to implement epigenetic biomarkers into better clinical management.

## Introduction

Cervical cancer (CC) is one of the most prevalent and lethal human malignancies worldwide. It is widely recognized that persistent infection with high-risk types of the human papillomavirus (HPV) leads to transformation from cervical intraepithelial neoplasia (CIN) to neoplasms (1, 2). Although improvements have been achieved in early cytomorphological screening, vaccines against HPV, advanced treatment strategies, the incidence and mortality have increased over the past decades, especially in developing countries (3, 4). In China, 140,000 new cases and 80,000 women deaths from CC occur annually, which affected youth trends more clearly (5). Consequently, better therapy and stratification of patients with CC at high risk of treatment failure remains a significant challenge, and there is urgently needed to identify promising diagnostic and prognostic biomarkers.

Complex and heterogeneous genomic aberrations and mutations induced by high-risk HPV contribute to the initiation and progression of CC (6–8). However, the association between high-risk HPV infection and DNA methylation has not been investigated. A growing number of studies suggest that epigenetic abnormalities such as aberrant DNA hypermethylation within gene promoters in various tumor entities, including CC (9, 10). For example, well-known tumor suppressors or tumor-related genes (*CDH1, CDKN2A, DAPK*, etc.) are silenced by promoter methylation in CC (11). High-risk HPV infection has been demonstrated to regulate DNA methylation in HPV-related cancers. Both E6 and E7 viral oncogene were reported to affect DNA methyltransferase activity, which increased our understanding of molecular principles underlying the pathogenesis of HPV-related cancers (12–14). In the past, an HPV-related gene promoter methylation signature of 5 genes (*ALDH1A2, OSR2, GRIA4, GATA4, *and* IRX4*) was identified in HPV-driven head and neck squamous carcinoma (HNSCC) by a genome-wide array approach (15). The significant correlation between promoter hypermethylation and suppressed gene expression indicated a vital role of candidate genes in HPV-related cancers.

The promising prediction value of HPV-related gene promoter methylation signature raised the attractive question of whether the gene signature could be employed as a reliable prognosticator for clinical staging and prognosis of CC. In the present study, HPV-related gene promoter methylation signature was comprehensively analyzed using multiple interactive platforms, including cBioPortal, GEPIA2, UALCAN (16–18). In addition, HPV-related methylation-based gene signature and clinical prognosis of patients with CC were examined in The Cancer Genome Atlas (TCGA) database (19). Finally, the correlation of ALDH1A2 protein expression and clinical prognostic value was confirmed in the CC patient cohort.

## Results

### Comprehensive Analysis of the HPV-Related Gene Signature in Multiple Cancer Types

**Figure 1:**
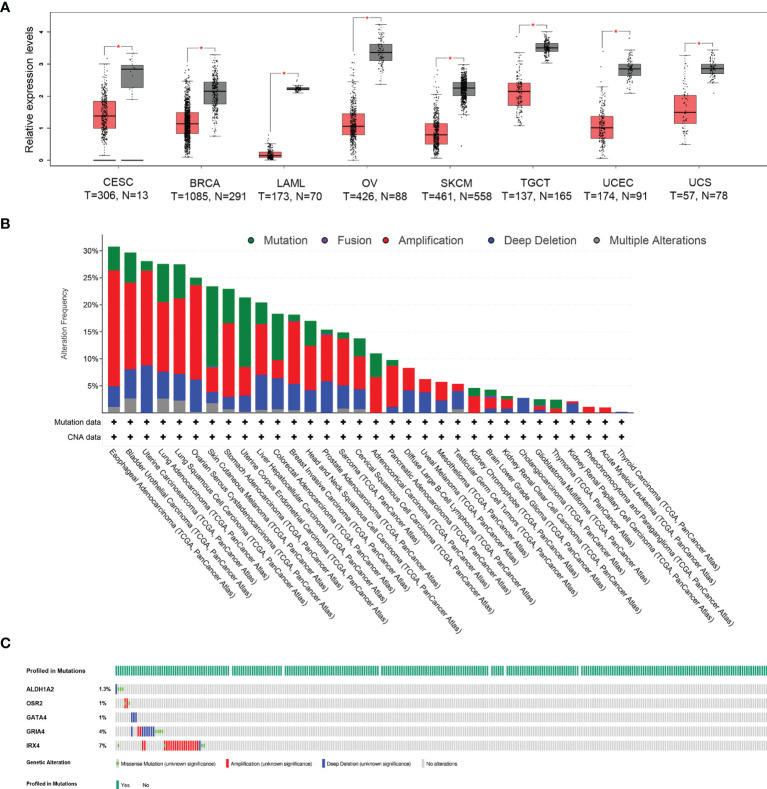
Different expression and mutational landscape of HPV-related gene signature in multiple TCGA-cohorts. **(A)** The significantly different expression profile of HPV-related gene signature between tumor and normal tissues (GTEx database) was identified in eight TCGA-cohorts, including Cervical squamous cell carcinoma and endocervical adenocarcinoma (CESC), Breast invasive carcinoma (BRCA), Acute Myeloid Leukemia (LAML), Ovarian serous cystadenocarcinoma (OV), Skin Cutaneous Melanoma (SKCM), Testicular Germ Cell Tumors (TGCT), Uterine Corpus Endometrial Carcinoma (UCEC), Uterine Carcinosarcoma (UCS), *P < 0.05. **(B)** Genetic alterations of HPV-related gene signature from 32 TCGA-cohorts were presented. **(C)** Oncoprint shows genetic alterations of the five-gene signature in the cohort of CESC.

To evaluate the differentially expressed HPV-related gene signature between tumor and normal tissues, the online web server GEPIA2 was applied to analyze the gene expression data from TCGA cohorts and GTEx projects. Differential signature score analysis revealed that the HPV-related gene signature was significantly reduced in the tumor tissues of eight cancer cohorts as compared to the expression levels in corresponding non-tumor tissues, including Cervical squamous cell carcinoma and endocervical adenocarcinoma (CESC), Breast invasive carcinoma (BRCA), Acute Myeloid Leukemia (LAML), Ovarian serous cystadenocarcinoma (OV), Skin Cutaneous Melanoma (SKCM), Testicular Germ Cell Tumors (TGCT), Uterine Corpus Endometrial Carcinoma (UCEC), Uterine Carcinosarcoma (UCS). In the cohort of CESC, 306 tumor tissues and 13 normal tissues were compared (**Figure 1A**). A pan-cancer analysis with 10,967 tumor samples in 32 TCGA cohorts revealed that a low frequency of *ALDH1A2* (1.4%), *OSR2* (5%), *GRIA4* (3%), *GATA4* (4%), and *IRX4* (5%) was genetically altered. The high frequency of genetic alterations in the five genes was observed in several cancers, including esophageal adenocarcinoma (30.77%), bladder urothelial carcinoma (29.68%), uterine carcinosarcoma (28.07%), lung adenocarcinoma (27.56%), lung squamous cell carcinoma (27.52%) (**Figure 1B**). 13.8% of 297 cervical squamous cell carcinoma cases revealed that genetic alterations in the five-gene signature with a low frequency of *ALDH1A2* (1.3%), *OSR2* (1%), *GRIA4* (4%), *GATA4* (1%), and *IRX4* (7%) (**Figure 1C**). Together, the gene expression level of HPV-related gene signature was significantly lower in cervical cancer as compared to the level of normal tissues. In contrast, a rather low frequency of genetic alterations was observed.

### Promoter Methylation of HPV-Related Gene Signature in Cervical Cancer

**Figure 2:**
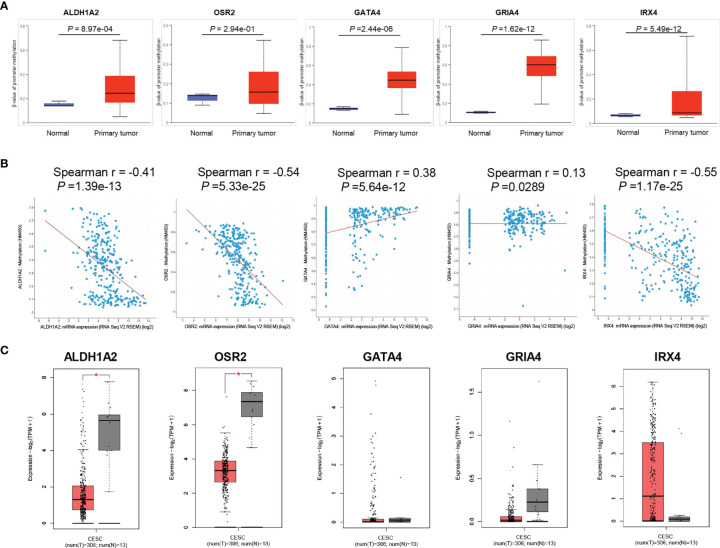
Promoter methylation analysis of HPV-related gene signature in TCGA-CESC cohort. **(A)** Box and whisker plots revealed methylation β-values of HPV-related gene signature between tumor and normal tissues, *P* values were presented for each gene. **(B)** Spearman correlation analysis between gene promoter methylation and relative transcript levels for each gene were performed by the cBioPortal tool, *P* values were presented for each gene. **(C)** The significantly different expression level of each gene was presented by boxplots, *P < 0.05.

**Figure 3:**
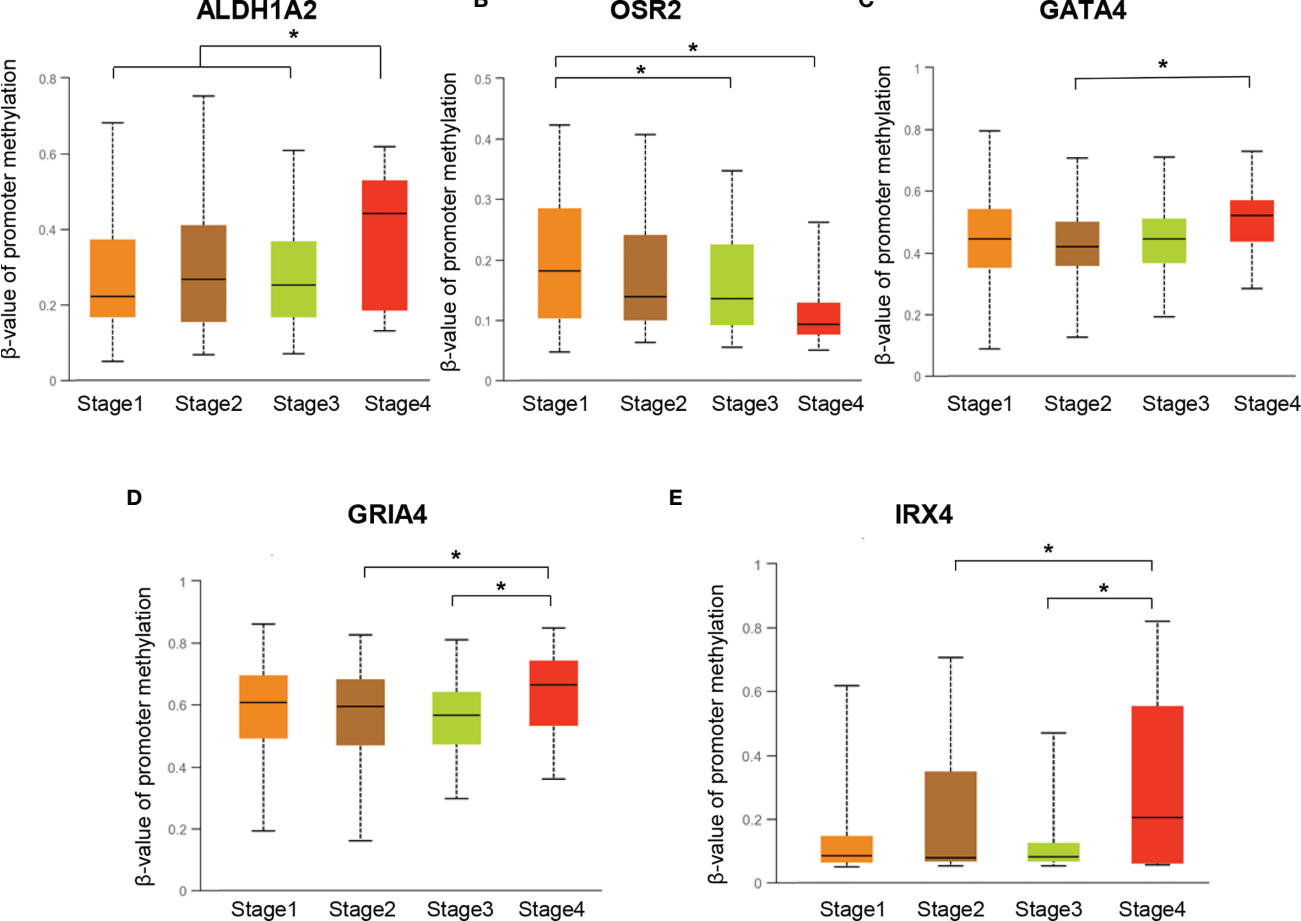
Promoter DNA methylation levels of ALDH1A2 **(A)**, OSR2 **(B)**, GATA4 **(C)**, GRIA4 **(D)**, and IRX4 **(E)** were compared among the patients with different clinical-stages, *P < 0.05.

To investigate the promoter methylation data of HPV-related gene signature in cervical cancer, we applied an interactive web-portal UALCAN to perform analyses for TCGA-CESC. Box whisker plots revealed that promoter DNA methylation levels (average beta values) of *ALDH1A2, GATA4, GRIA4*, and *IRX4* were significantly elevated in primary tumors as compared to normal tissues (**Figure 2A**). It is generally accepted that gene promoter hypermethylation may downregulate transcription of the affected gene. We observed a significant inverse correlation between gene promoter methylation and relative transcript levels for *ALDH1A2, OSR2*, and *IRX4* (**Figure 2B**). Differential expression analysis revealed that the levels of *ALDH1A2* and *OSR2* were significantly reduced in the tumor tissues as compared to the normal tissues (**Figure 2C**). Thus, our findings suggest that *ALDH1A2*, *OSR2* gene transcription is regulated by gene promoter methylation, which is suppressed in the primary tumors. In addition, promoter DNA methylation levels (average beta values) of *ALDH1A2, OSR2, GATA4, GRIA4*, and *IRX4* were compared among the patients with different clinical stages. Box whisker plots showed that promoter DNA methylation levels of *ALDH1A2* in tumor clinical stage IV were significantly elevated than any other subgroup with stage I/II/III (**Figure 3A**). Concerning average beta values of *GATA4, GRIA4*, and *IRX4*, the patients with tumor stage IV had the highest level as compared to the subgroup patients with stage I/II/III tumor (**Figures 3C–E**). By contrast, *OSR2* promoter DNA methylation level in patients with stage I tumors was significantly higher as compared to the patients with stage III/IV tumors (**Figure 3B**). In summary, promoter methylation of HPV-related gene signature is significantly associated with the CC clinical stage.

### HPV-Related Methylation-Based Gene Signature Was Significantly Associated With the Clinical Prognosis of Patients With Cervical Cancer

**Figure 4:**
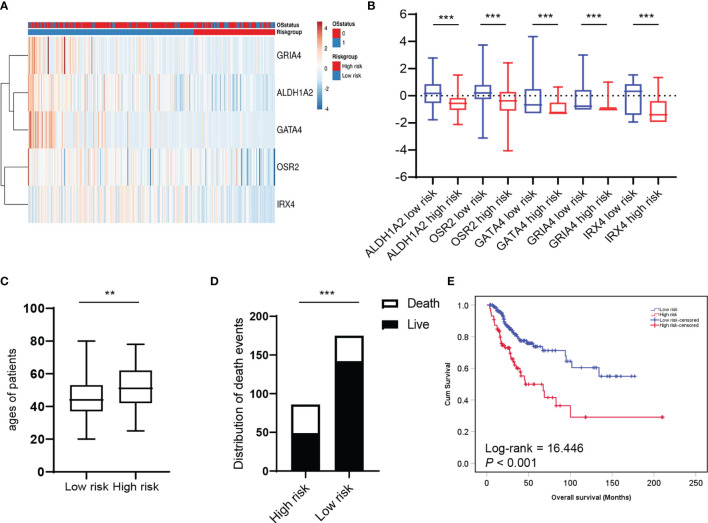
HPV-related methylation-based gene signature is associated with the clinical prognosis of patients with cervical cancer. **(A)** Heatmap represented HPV-related gene signature expression values (rows) and tumor samples (columns) in low and high-risk groups by supervised clustering. **(B)** Box and whisker plots of HPV-related gene signature expression values were compared between two risk groups by *t*-test. The average ages **(C)** and the distribution of death events **(D)** between two risk groups were compared. **(E)** Overall survival of two risk groups was evaluated by Kaplan–Meier survival plot and Log-Rank test. **P < 0.01, ***P < 0.001.

Next, we asked whether HPV-related methylation-based gene signature is associated with the clinical prognosis of patients with cervical cancer. A heat map based on *ALDH1A2*, *OSR2*, *GATA4*, *GRIA4*, and *IRX4* transcript levels were generated by the *SurvExpress* web tool, and two clusters with either low or high prognostic risk were defined by prognostic index (PI) and Cox fitting (20, 21) (**Figure 4A**). The expression level of the five genes was compared between the high-risk group (n=86) and the low-risk group (n=175), which revealed the levels of the five genes were significantly reduced in the high-risk group (**Figure 4B**). As expected, the age of patients and the number of death events in the high-risk group are significantly higher as compared to the low-risk group (**Figures 4C, D**). Chi-square analysis showed the low-risk group is significantly associated with younger age (*P*=0.002) and lower T status (p=0.006) (**Supplementary Table S1**). Furthermore, the Kaplan-Meier plot and Log-Rank analysis exhibited a significant difference in overall survival between the high and low-risk groups (**Figure 4E**; Log-rank = 16.446, *P* < 0.001). In summary, these findings demonstrated that HPV-related methylation-based gene signature predicts the clinical outcome for CC patients.

### The Protein Levels of ALDH1A2 in Cervical Cancer Were Correlated With Clinical Outcomes

**Figure 5:**
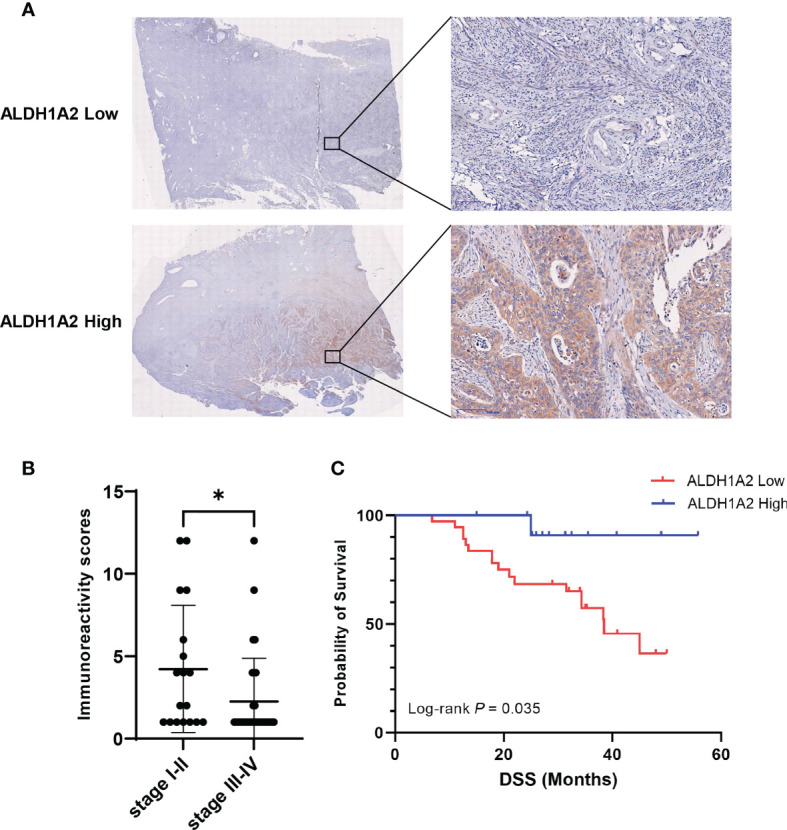
Correlation between ALDH1A2 protein levels and clinical prognosis of patients with cervical cancer. **(A)** Representative pictures of IHC stained primary tumor sections with different expression of ALDH1A2 protein levels (brown signal). Counterstaining with hematoxylin. Scale bars:100μm. **(B)** Immunoreactivity scores were compared between the low clinical stage (I/II) (n=18) and the high clinical stage (III/IV) (n=32). Bars depict mean values ± SEM of IRS, *P < 0.05. **(C)** Disease-specific survival of patients with cervical cancer separated by high versus low ALDH1A2 expression. *P*-value was derived by log-rank test.

To confirm the prognostic value of the candidate gene *ALDH1A2* in the protein level, we determined ALDH1A2 protein levels by immunohistochemical (IHC) staining in primary tumor tissues from patients with cervical cancer, and we found the heterogeneous cytoplasmic expression of ALDH1A2 in tumor tissues (**Figure 5A**). The patients were divided into two subgroups depending on different immunoreactivity scores (IRS) with ALDH1A2^high^ (n = 13) and ALDH1A2^low^ (n = 37) for further analysis. The patients with clinical stage I/II had a statistically higher IRS of ALDH1A2 as compared to the patients with stage III/IV (**Figure 5B**), which was in line with our *in silico* analysis findings. Survival analysis revealed that patients with ALDH1A2^low^ expression tumors had a significantly worse disease-specific survival (DSS) as compared to the patients with ALDH1A2^high^ expression pattern (**Figure 5C**). Taken together, our results showed low expression of ALDH1A2 serves as an unfavorable risk factor and a prognosticator to identify patients with cervical cancer at high risk for treatment failure.

## Discussion

Persistent high-risk HPV infection drives tumorigenesis in various types of human malignancies, including cervical, oropharyngeal, anal, and vulvar carcinomas (22–24). Although HPV-related tumors arise in several different sites, they share many common genetic and epigenetic events. As one of the most significant epigenetic modifications, aberrant DNA methylation is a fundamental epigenetic event for HPV-related carcinogenesis. DNA methylation signatures have been proven to predict the clinical prognosis in a variety of cancers (25–29). In the past, HPV-related alterations in the DNA methylome of patients with oropharyngeal squamous cell carcinoma (OPSCC) were screened, and a methylation score based on the five-gene promoter methylation pattern was identified for a reliable prognostic factor (15). The significant correlation between promoter hypermethylation and suppressed gene-level indicated the critical function of candidate genes in HPV-driven carcinogenesis and the response to treatment.

In this study, we performed the first comprehensive analysis of HPV-related gene promoter methylation signature of five-gene (*ALDH1A2, OSR2, GRIA4, GATA4*, and *IRX4*) in TCGA database. We found the total expression level of HPV-related gene signature was significantly lower in cervical cancer as compared to the level of normal tissues, whereas a rather low frequency of genetic alterations was observed. In addition, promoter DNA methylation levels (average beta values) of *ALDH1A2*, *GATA4*, *GRIA4*, and *IRX4* were significantly elevated in primary cervical carcinoma as compared to normal tissues. As expected, differential expression analysis revealed that the levels of *ALDH1A2* and *OSR2* were significantly reduced in the tumor tissues as compared to the normal tissues. Elevated promoter DNA methylation level of *ALDH1A2* was observed in the advanced clinical stage, suggesting that *ALDH1A2* gene expression was associated with the clinical outcome in cervical cancer patients. Despite aberrant promoter hypermethylation of *ALDH1A2*, *OSR2*, *GATA4*, and *GRIA4* have already been reported in several human tumor cell lines or tumor tissues. To the best of our knowledge, there is no study focusing on the methylation signature of five genes in cervical cancer. To highlight the prognostic value of HPV-related methylation signature, a risk-scoring model was established, and the risk scores were calculated based on *ALDH1A2*, *OSR2*, *GATA4*, *GRIA4*, and *IRX4* transcript levels as well as the regression coefficient of each gene. We found the ages, cancer-related death events, T status were significantly different between patients in high-risk and low-risk subgroups. We also observed the significantly different overall survival between two risk groups, which was evaluated by Kaplan-Meier analysis and log-rank test. A previous study reported that CC patients could be divided into three heterogeneous clusters base on HPV-related methylation sites, which supports that DNA methylation sites can serve as biomarkers for subgroup identification and prognostic risk stratification (30). Despite these promising achievements, heterogeneous clusters based on the HPV-related methylation sites turned out to be a limitation for the potential translation into clinical practice.

So far, our study investigated the one gene-encoded protein expression, *ALDH1A2*, in the primary tumor tissues from patients with cervical carcinoma. We excluded the *GATA4*, *GRIA4*, and *IRX4* genes due to no significant difference between the tumor tissues and the normal tissues in cervical carcinoma based on the TCGA-CESC data. Promoter DNA methylation levels (average beta values) of *OSR2* revealed no significant changes between primary tumors and normal tissues. Moreover, low cancer specificity of *OSR2* was found based on the literature review. Our presented data indicate that the expression and function of the *ALDH1A2* gene are more relevant for the clinical prognosis of CC patients. The gene *ALDH1A2* encodes aldehyde dehydrogenase one family member A2 (ALDH1A2), which is a rate-limiting enzyme involved in the cellular synthesis of retinoic acid. Previous studies have demonstrated ALDH1A2 to be a candidate tumor suppressor in several human malignancies, including prostate, head and neck, ovarian cancer (15, 31–33). However, it had not been determined whether reduced ALDH1A2 protein expression is relevant to the prognosis of CC patients. Therefore, ALDH1A2, promisingly suitable for the clinical prognosis of CC patients, was selected to determine protein levels by immunohistochemical (IHC) staining in primary tumor tissues from patients with cervical cancer. In line with the previous studies, our results showed that the expression of ALDH1A2 at an advanced stage is lower than that at an early tumor stage. Furthermore, a low expression level of ALDH1A2 protein is significantly associated with unfavorable clinical outcomes of CC patients, which indicates that abnormal expression of ALDH1A2 might play a critical role in CC carcinogenesis and prognostic value. However, the detailed mechanism underlying the association between *ALDH1A2* gene methylation and the progression of CC requires further investigation. Moreover, we will certainly increase the number of patient cohorts and will investigate the protein expressions of four other HPV-related tumor suppressor genes in cervical cancer.

## Conclusion

In summary, our study provides compelling evidence that HPV-related gene promoter methylation and expression successfully classify CC patients into high-risk and low-risk groups with significant differences in prognosis. The five methylated-differentially expressed genes signature was promising for the translation from bench to bedside to evaluate the prognosis of CC patients, which offers a novel strategy to identify patients at high risk for treatment failure. Moreover, encoded proteins regulated by the gene promoter methylation might serve as reliable markers for early diagnosis, risk stratification, and innovative cancer treatment.

## Materials and Methods

### Bioinformatics Analysis

Mutation and CNA analyses were conducted on the TGCA PanCanAtlas datasets using the cBioPortal database version 3.6.12 (Center for Molecular Oncology at MSK, New York, NY, USA). DNA methylation data and gene expression data from TCGA-CESC were accessed through cBioPortal tool. Correlation analysis was performed between methylation beta values and log-base-2-transformed gene expression data with Pearson’s correlation with a Bonferroni correction to the *P*-values. Expression in various cancers and their normal tissue counterparts were analyzed using the Gene Expression Profiling Analysis (GEPIA) (Beijing, China). GEPIA2 provides RNA sequencing data from TCGA of tumor samples with paired adjacent TCGA and Genotype-Tissue Expression (GTEx) normal tissue samples. TCGA and GTEx RNA-Seq expression datasets in GEPIA are based on the UCSC (University of California, Santa Cruz) Xena project (34), which are recomputed based on a uniform bioinformatic pipeline to eliminate batch effects. To compare expression data, data are normalized by quantile-normalization or other two additional normalization strategies. UALCAN database (http://ualcan.path.uab.edu) was used to compare the promoter methylation of HPV-related gene signature between normal and primary CC tissues, as well as methylation beta values of each gene among different tumor stages.

We downloaded the five genes expression profile of TCGA-CESC from cBio Cancer Genomics Portal (http://cbioportal.org) in December 2020. Low-risk and high-risk groups of patients with CC were generated depending on the prognostic index (PI) and Cox fitting. A heat map representation of the five genes expression values was made by supervised clustering with *ClustVis* (21), a freely available web server at http://biit.cs.ut.ee/clustvis/.

### Patient Tissue Specimens

Patients with primary CC diagnosed and treated between 2015 and 2020 were comprised in the retrospective study cohort. Tumor tissue sections from formalin-fixed paraffin-embedded (FFPE) were obtained at the Department of Pathology, The First Affiliated Hospital of University of Science and Technology of China after approval by the ethics committee. Biopsies of non-surgically treated patients, as well as samples of patients who underwent tumor surgery, were included. All subjects gave written informed consent for data collection as it is a standard procedure in our department. Patients with suspicious clinical findings who underwent diagnostic endoscopy and/or patients before tumor surgery with a histologically confirmed diagnosis of CC were asked to consent. The protocol was approved by the Ethics Committee of the First Affiliated Hospital of USTC (Ethic No 2021/BLK02) in accordance with the declaration of Helsinki. Experimental treatment procedures were not part of this study. The patients were treated according to the guidelines for cervical cancer. The final analysis was based on 50 patients with CC (**Supplementary Table S2**). Clinical and therapeutic follow-up of the cohort was assessed retrospectively. The cohort did not include recurrences or specimens from surgery.

### Immunohistochemical Staining and Pathological Scoring

Immunohistochemical staining was carried out in paraffin-embedded tissue from patients with primary CC. Tissue sections were deparaffinized and rehydrated by the following our previous study (35) and incubated with primary antibody ALDH1A2 (1:500, HPA010022, Sigma-Aldrich) at 4°C overnight. Tissue sections were incubated with a biotinylated anti-rabbit secondary antibody (Boster) for 2 h. Streptavidin-HRP (Boster) and Peroxidase Substrate (DAB) solution (MXB Biotechnologies, Fuzhou, China) were added to the tissue sections for signal development. Tissue slides were scanned using the DMS-10 Scan System (D-metrix, Suzhou). Three experienced pathologists analyzed scanned slides by using the D-metrix Viewer software (version 1.0.0). Semiquantitative analysis was performed according to the number of stained tumor cells (score A; 1, no positive cells; 2, less than 33% positive cells; 3, between 34% and 66% positive cells; 4, more than 66% positive cells), and according to the staining intensity (score B; 1, no staining; 2, weak staining; 3, moderate staining; 4, high staining). Both values were multiplied to calculate the final immunoreactivity score (IRS) for defined subgroups (final expression scores ranged 1-16).

### Statistical Analysis

All statistical analysis was done using GraphPad Prism version 9.1 (GraphPad Software, USA) and IBM SPSS Statistics version 22. Differences of clinicopathological features between the low-risk and high-risk groups were compared using Chi-square Test or Fisher’s exact test. Significance of the difference was calculated by student’s *t*-test. Disease-specific survival (DSS) was estimated as the time from the date of primary tumor diagnosis to the date of CC-related death within the follow-up period (events) or to the date of CC unrelated death or without progression (censored). Survival difference was assessed by Kaplan-Meier plot and Log-Rank analysis. A *P*-value <0.05 was considered statistically significant.

## Data Availability Statement

Publicly available datasets were analyzed in this study. This data can be found here: http://cbioportal.org.

## Ethics Statement

The studies involving human participants were reviewed and approved by Ethics Committee of the First Affiliated Hospital of USTC (Ethic No 2021/BLK02). The patients/participants provided their written informed consent to participate in this study.

## Author Contributions

CR and S-LW conceived and guided the study. RZ, ZC, and Z-RX performed the experiments. RZ, ZC and CR analyzed and interpreted the data. RZ and CR contributed to the writing and data presentation of the manuscript. All authors contributed to the article and approved the submitted version.

## Funding

This study was supported by the grants from the Natural Science Foundation of Jiangsu Province (No. BK20200878), the Natural Science Foundation of the Jiangsu Higher Education Institutions of China (No. 20KJB310014), and the Priority Academic Program Development of Jiangsu Higher Education Institutions (PAPD).

## Conflict of Interest

The authors declare that the research was conducted in the absence of any commercial or financial relationships that could be construed as a potential conflict of interest.

## Publisher’s Note

All claims expressed in this article are solely those of the authors and do not necessarily represent those of their affiliated organizations, or those of the publisher, the editors and the reviewers. Any product that may be evaluated in this article, or claim that may be made by its manufacturer, is not guaranteed or endorsed by the publisher.
