# Supplementary material for: HPV-Related Promoter Methylation-Based Gene Signature Predicts Clinical Prognosis of Patients With Cervical Cancer
Source: Front Oncol. 2021 Oct 21;11:753102. doi: 10.3389/fonc.2021.753102 (PMC8566918; doi:10.3389/fonc.2021.753102)
Supplement: Supplementary file 1 [file Table_1.docx]

Supplemental **Table S1**. Association between two risk groups and clinicopathological features in TCGA-CESC cohort (n=261)

| **Clinicopathological Features** | **Low Risk group** | **High Risk group** | ***P* -Value** |
| --- | --- | --- | --- |
| Age(years) |  |  | **0.002** |
| < 48 | 108 | 36 |  |
| ≥48 | 67 | 50 |  |
| TNM status |  |  | **0.006** |
| T1 | 90 | 33 |  |
| T2-T4 | 47 | 39 |  |
| N.A. | 38 | 14 |  |
| N0 | 78 | 35 | 0.521 |
| N+ | 37 | 13 |  |
| N.A. | 60 | 38 |  |
| M0 | 64 | 33 | 0.092 |
| M+ | 10 | 1 |  |
| N.A. | 101 | 52 |  |
| Pathological Grade |  |  | 0.622 |
| G1/G2 | 92 | 46 |  |
| G3 | 69 | 30 |  |
| missing | 14 | 10 |  |

**Supplemental Table S2**. Clinical features of CC patients cohort (n=50)

| **Clinicopathological Features** | **Category** | **N** | **%** |
| --- | --- | --- | --- |
| Age(years) | < 47.4 | 16 | 32% |
|  | ≥47.4 | 34 | 68% |
| T | T1-T2 | 18 | 36% |
|  | T3-T4 | 32 | 64% |
| N | N0 | 38 | 76% |
|  | N+ | 12 | 24% |
| M | M0 | 50 | 100% |
|  | M+ | 0 | 0% |
| Pathological Grade | G1/G2 | 19 | 38% |
|  | G3 | 31 | 62% |
| Alcohol | Yes | 0 | 0% |
|  | No | 50 | 100% |
| Tobacco | Yes | 0 | 0% |
|  | No | 50 | 100% |
